# The Opportunistic Pathogen *Listeria monocytogenes*: Pathogenicity and Interaction with the Mucosal Immune System

**DOI:** 10.4061/2010/704321

**Published:** 2010-07-14

**Authors:** Markus Schuppler, Martin J. Loessner

**Affiliations:** Institute of Food, Nutrition and Health, ETH Zurich, Schmelzbergstraße 7, 8092 Zurich, Switzerland

## Abstract

*Listeria monocytogenes *is an opportunistic foodborne pathogen causing listeriosis, an often fatal infection leading to meningitis, sepsis, or infection of the fetus and abortion in susceptible individuals. It was recently found that the bacterium can also cause acute, self-limiting febrile gastroenteritis in healthy individuals. In the intestinal tract, *L. monocytogenes *penetrates the mucosa directly via enterocytes, or indirectly via invasion of Peyer's patches. Animal models for *L. monocytogenes *infection have provided many insights into the mechanisms of pathogenesis, and the development of new model systems has allowed the investigation of factors that influence adaptation to the gastrointestinal environment as well as adhesion to and invasion of the intestinal mucosa. The mucosal surfaces of the gastrointestinal tract are permanently exposed to an enormous antigenic load derived from the gastrointestinal microbiota present in the human bowel. The integrity of the important epithelial barrier is maintained by the mucosal immune system and its interaction with the commensal flora via pattern recognition receptors (PRRs). Here, we discuss recent advances in our understanding of the interaction of *L. monocytogenes *with the host immune system that triggers the antibacterial immune responses on the mucosal surfaces of the human gastrointestinal tract.

## 1. Introduction

*Listeria monocytogenes* is a Gram-positive foodborne pathogen that is ubiquitously found in diverse environments such as soil, water, various food products, animals, and humans [1]. Infection by *Listeria monocytogenes* occurs almost exclusively after ingestion of contaminated food. Because the bacteria are readily inactivated at pasteurization temperature, the main source of infection represents contaminated raw food that is subjected to minimal further processing, such as soft cheeses, frankfurters, pâtés, vegetables and postprocessed contaminated milk products [2]. In individuals with impaired cell-mediated immunity such as neonates, pregnant woman, elderly persons, and immunocompromised patients suffering from transplantation events, the bacterium may cause mother-to-fetus infections, septicemia, or meningoencephalitis. Listeriosis is relatively rare and annual incidence is decreasing; in the United States from 7.7 cases per million population in 1990 to 3.1 cases per million population in 2003. In France, the incidence of listeriosis declined from 4.5 cases per million population in 1999–2000 to approximately 3.4 in 2002–2003 [3]. Although the incidence is low, the high mortality rates (about 30%) associated with listeriosis make *L. monocytogenes* one of the most deadly human food-borne pathogens. In contrast to the severe invasive disease recent outbreaks demonstrated that infection of healthy individuals with *L. monocytogenes *often leads to the development of a febrile gastroenteritis [4].

The organisms are well adapted to the conditions in the gastrointestinal tract and pursue different strategies to counteract changes in acidity, osmolarity, oxygen tension, or the challenging effects of antimicrobial peptides and bile. The finding that the bacteria are able to colonize and persist in the gallbladder [5] suggests the occurrence of long-term and chronic infections and demonstrates the ability of pathogenic *Listeria* to survive within the various microenvironments of the gastrointestinal tract. 

Although other animals, such as guinea pigs, seem to be better suited to study the immune response to *L. monocytogenes* mice have been proven the most useful model for immunological studies due to availability of knock-out mice deficient in specific genes. Hence, most of our knowledge of how the immune system functions has been learned from experimental infections of mice using *L. monocytogenes* and the subsequent analysis of the innate and adaptive immune responses [6]. The molecules that function as pattern recognition receptors (PRRs) on epithelial cells, macrophages, and dendritic cells (DCs), thereby triggering the innate immune system after contact to bacterial pathogens, comprise Toll-like receptors (TLRs) and nucleotide-binding oligomerization domain (NOD)-like receptors (NLRs). The recognition of pathogen-associated molecular patterns (PAMPs) by the PRRs on mucosal cells drives the activation of subsequent signaling cascades including NF-*κ*B, interferon (IFN) response factors (IRFs), activator protein 1 (AP1), and mitogen-activated protein kinases that promote the induction of proinflammatory cytokines and antimicrobial peptides, as well as the maintenance of epithelial barrier function and epithelial cell proliferation [7]. The vast amount of knowledge that has been gathered through proteomic and transcriptomic approaches makes *L. monocytogenes* one of the best-studied bacterial pathogens for investigations on the interplay of intracellular pathogens and the intestinal immune system. In this paper, we focus on the recent developments in the analysis of the interaction between *L. monocytogenes* and the mucosal immune system of the host gastrointestinal tract.

## 2. Adaptation of *Listeria monocytogenes* to the Conditions of the GI Tract

Along the gastrointestinal tract *L. monocytogenes* cells have to face a hostile environment characterized by altered osmolarity, low oxygen pressure, low pH, and presence of bile. In a recent article Lungu et al. [8] reconsider growth, survival, proliferation and pathogenesis of *L. monocytogenes* under low oxygen or anaerobic conditions. Other studies have shown that *L. monocytogenes* is able to launch acid resistance systems to respond to the low pH conditions in food or in the host [9, 10]. In *L. monocytogenes,* the glutamate decarboxylase (GAD) system confers resistance to acidic environments. Cotter and colleagues demonstrated that the expression of the GAD system correlates directly with acid tolerance of *L. monocytogenes* and is an absolute requirement for survival during the transmission of the stomach [9]. This system has already been associated with acid resistance in many other bacteria that need to transit the stomach before they reach their site of infection. The GAD system leads to intracellular consumption of protons by irreversible decarboxylation of extracellularly sourced glutamate and subsequent export of gamma-aminobutyrate (GABA) via a glutamate:GABA antiporter. Surprisingly, it was shown that acid-adapted *L. monocytogenes *were more successful in entering and proliferating in Caco-2 cells in contrast to cells that were not previously exposed to acid-stress [11].

*L. monocytogenes* encounters changes in the osmolarity not only in the gastrointestinal tract of its host but also in the food industry, as a preservation method or in their environmental niches. The strategy that *Listeria* and also other bacteria apply to respond to conditions of elevated osmolarity is the cytoplasmic accumulation of compatible solutes or osmolytes (reviewed by Sleator and Hill [13]). The finding that *L. monocytogenes* is able to colonize the gall bladder of infected mice demonstrates the organisms abilities to tolerate high concentrations of bile stored in this compartment [14]. The genes conferring the principal bile-resistance to *L. monocytogenes* are BSH and BilE [5]. This resistance has important consequences as Dussurget et al. [15] reported that bile salt hydrolase activity is essential for *L. monocytogenes* pathogenesis. Furthermore, carnitine uptake by the pathogen is essential for survival in the small intestine and transient colonization of the murine gastrointestinal tract [16, 17].

In the context of adaptation of *L. monocytogenes* to the conditions along the GI tract, the transcription factor Sigma^B^ (*σ* ^B^) was identified as the key factor that triggers the manifold adaptation mechanisms. The crucial role that the *σ* ^B^ protein plays in acid-tolerance was identified by Wiedmann et al. [18], whereas Becker et al. [19] independently identified *σ* ^B^ as the relevant factor for adaptation of *L. monocytogenes* to alterations in osmolarity and temperature. Also, the genes for BSH and BilE responsible for bile-tolerance are preceded by *σ* ^B^ dependent promoter sites [5, 20].

**Figure 1:**
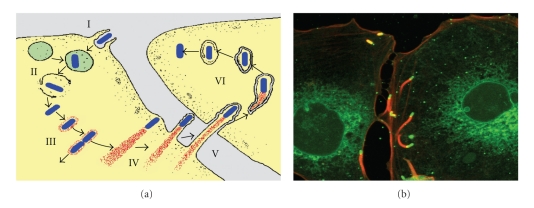
Stages in the intracellular life cycle of *L. monocytogenes*. The cartoon (a) sketches the different stages of *L. monocytogenes* infection: (I) cell entry mediated by invasion factors InlA or INLB, (II) escape from phagolysosom by LLO and PlcA, (III) actin recruitment and replication, (IV) intracellular movement due to polarized actin-polymerization mediated by ActA, (V) cell-to-cell spread by formation of listeriapods, and (VI) subsequent lysis of the two-membrane vacuole by LLO and PlcB. Modified from Tilney & Portnoy [12]. The fluorescence image (b) shows the intracellular movement and cell-to-cell spread of *L. monocytogenes* cells (green) driven by the polarized polymerization of actin tails (red).

In their study, Kazmierczack et al. [21] identified fifty-five genes showing a statistically significant *σ* ^B^ dependent expression after exposure of *L. monocytogenes* to osmotic stress. In a more recent study by Hain et al. [22], the authors report a significantly higher number of genes that were under the regulation of *σ* ^B^. They found 111 genes under negative control of *σ* ^B^ and 105 genes that showed a positive *σ* ^B^ dependent regulation. In *L. monocytogenes σ* ^B^ contributes to both stationary- and exponential-phase acid resistance, whereas in *L. innocua* acid resistance is conferred by *σ* ^B^ only during the exponential-phase of growth [23]. Thus, the function of *σ* ^B^ seems to be strain and species dependent within the genus *Listeria*. Moreover, it turned out that different serotypes of *L. monocytogenes *reveal differences in their dependency on a functional *σ* ^B^ regulon [24]. As a consequence, the resulting variations in environmental stress resistance may offer an explanation for the reported differences in the virulence traits and the survival capabilities in the host and in food for different *L. monocytogenes *strains [18]. The obtained results indicate that *σ* ^B^ contributes to *L. monocytogenes* survival not only in environmental niches but also inside the host [25, 26]. This is emphasized by the finding that *σ* ^B^ is required for the expression of the manifold genes that are important for the survival of *L. monocytogenes *within the GI tract of a host. In fact, the *σ* ^B^ regulon comprises important virulence genes, encoding virulence factors such as internalin A and B [21, 22]. In murine and guinea pig models, loss of *σ* ^B^ function has been shown to result in decreased virulence of *L. monocytogenes *after oral infection but not during systemic infection [27]. Furthermore, *L. monocytogenes *show an up-regulation of the *sigB* gene during passage of the mouse GI tract [5]. Hence, the alternative sigma factor *σ* ^B^ represents a crucial prerequisite for the successful infection of a host by *Listeria monocytogenes *via the GI route (Figure 1). 

## 3. Gastroenteritis due to *Listeria monocytogenes*

Listeriosis is a severe foodborne disease characterized by bacteremia and meningoencephalitis in individuals with impaired cell-mediated immunity, including neonates, pregnant woman, elderly persons, and immunosuppressed patients. The incidence of listeriosis is rather low, compared to other common foodborne pathogens such as *Campylobacter* species, *Salmonella* species, *Shigella* species, and* Vibrio* species. However, the outcome is much more severe and often fatal. In fact, it represents one of the most deadly bacterial infections due to its high mean mortality rate of 20%–30%, despite early antibiotic treatment [28]. Ingestion of food contaminated with* L. monocytogenes* is the usual mode of transmission leading to listeriosis. Although many patients experience diarrhea antecedent to the development of bacteremia or meningoencephalitis due to *L. monocytogenes *infection, it was only recently that convincing evidence was obtained that *L. monocytogenes* can cause self-limiting, febrile gastroenteritis in healthy persons [4]. At least seven outbreaks of foodborne gastroenteritis due to *L. monocytogenes *infection have been reported over the last 20 years (Table 1). First evidence was obtained in 1989 when the *L. monocytogenes *strains isolated from blood samples of two febrile pregnant women and those from the stool samples of a person with diarrhea were shown to be identical [29]. All three patients attended the same party, and a total of 17 of the 36 attendees reported gastrointestinal complaints. More convincing evidence that *L. monocytogenes* could cause self-limiting gastroenteritis came from an outbreak of febrile gastroenteritis that was associated with the consumption of contaminated chocolate milk [30]. A total of 45 out of 60 persons who drank chocolate milk served in the course of a picnic developed the symptoms, and identical strains were obtained from 14 symptomatic patients, from unopened cartons of the chocolate milk, as well as from the environment of the dairy that supplied the milk [30]. One of the largest documented outbreaks of febrile gastrointestinal illness comprised 292 persons who had been hospitalized after eating in the cafeteria of two primary schools in northern Italy in 1997. The contaminated food was prepared by the same caterer and cultures from 123 stool samples and 1 blood sample from the hospitalized patients turned out to be indistinguishable to strains isolated from the food, and environmental specimens of the catering plant [31]. Therefore, *L. monocytogenes *should be considered to be a possible etiologic agent in outbreaks of febrile gastroenteritis when routine cultures fail to yield a pathogen. 

Common symptoms observed in the effected patients included fever, watery diarrhea, nausea, headache, and pain in joints and muscles. The mechanism by which *L. monocytogenes* causes diarrhea is not entirely clear. However, it is likely that it is the result of direct invasion of the epithelial cells of the intestinal mucosa, as it is not known that *L. monocytogenes *produces enterotoxins [4]. The observed symptoms like fever as well as occasionally bloody diarrhea and bacteremia further support the hypothesis that diarrhea results from direct invasion of *L. monocytogenes* to the intestinal mucosal epithelium. The observation that *L. monocytogenes* could cause self-limiting, febrile gastroenteritis demonstrates that the pathogen induces mucosal inflammation after entering the host intestinal mucosa.

## 4. Adherence and Invasion of the Gastrointestinal Epithelium

There exist two principle mechanisms by which *L. monocytogenes* can enter into the host through the intestinal mucosa. The first route is direct invasion of the enterocytes lining the absorptive epithelium of the microvilli, leading to infection of the intestinal cells. This entry mechanism occurs only in humans and some susceptible animals (e.g., guinea pigs) that also express the correct isoform of the receptor molecules necessary for recognition by the *Listeria* invasion molecules, termed internalins [32]. The second entry pathway is translocation across the M-cells of Peyer's patches [33]. This mechanism occurs also in hosts that do not express susceptible isoforms of the receptor molecules, such as mice and rats, and represents an unspecific mechanism as nonpathogenic species such as *L. innocua* or *Bacteroides thetaiotaomicron*, a prominent gut symbiont, are translocated equally. However, the latter mechanism seems to be less efficient than direct invasion of enterocytes [34]. 

As a first step towards invasion of the gastrointestinal epithelium the bacteria need to adhere to the surface of the epithelial cells. To enable contact with the epithelium underlying the mucus layer as the site of invasion many bacteria produce mucinases. This is not the case for* L. monocytogenes* which does not produce mucinases but a number of surface proteins that can bind to a specific type of human mucin [35]. Interaction with the human Muc2 isoform occurs trough the internalin proteins InlB, InlC, and InlJ. This initial interaction is thought to be an important prerequisite for the subsequent events leading to adherence and invasion of the epithelial layer [28, 36]. For various gastrointestinal pathogens, it is known that they use their flagellar structures not only as effectors of motility but also as adhesins or as a secretion apparatus. This is also not the case for *L. monocytogenes *which uses flagella simply for motility thereby increasing the efficacy of host invasion [37, 38].

The initial interaction of internalins with Muc2 seems not to be sufficient and further expression of proteins is necessary to warrant adherence of the pathogen to the epithelium. *Listeria* adhesion protein (LAP) was shown to bind to the host cell heat-shock protein 60 [39] and a specific fibronectin-binding protein (FbpA) of *L. monocytogenes* was identified to interact with cell surface fibronectin in the murine model [40].

Central for the pathophysiology of *Listeria monocytogenes* in the gastrointestinal tract is the ability to cross the intestinal barrier through invasion of enterocytes. This important event is promoted by internalin A (InlA), whereas internalin B (InlB) seems to play no direct role in invasion of cells of the gastrointestinal epithelial layer. Instead, InlB is known to mediate the invasion of hepatocytes and is required for the infection of the fetoplacental unit [46, 47]. The cellular receptor for InlA is human E-cadherin, a protein expressed at the basolateral surface of polarized enterocytes that was identified by affinity chromatography on an InlA-column [48]. The InlA E-cadherin interaction is species-specific, and was shown to rely on a single amino acid residue in the E-cadherin molecule, which is prolin in permissive species such as humans, and glutamic acid in nonpermissive species such as the mouse [34]. Although the E-cadherin of mouse and human show about 85% similarity, InlA does not interact with mouse E-cadherin. For this reason, mice are not an appropriate experimental model for oral infection with *L. monocytogenes* and the investigation of the pathogenic events that enable the organisms to penetrate the intestinal mucosa after ingestion of contaminated food. Consequently, the necessity for further animal models for human listeriosis led to the identification of two novel and complementary animal models. While gerbils turned out to be a natural host for *L. monocytogenes*, a transgenic mouse line was developed that features expression of human E-cadherin in enterocytes [49]. Use of this animal model conclusively demonstrated the role of InlA for crossing the intestinal barrier [34], and the essential and interdependent roles of InlA and InlB in feto-placental listeriosis [46]. The detailed molecular mechanisms of InlA mediated cell entry have been described and reviewed elsewhere [1, 50–53]. 

Upon uptake, the intracellular pathogen appears surrounded by the membranes of the phagocytic vacuole. Different phospholipases (PI-PlcA and PI-PlcB) are activated by a metalloprotease (Mpl), and cooperate with the pore-forming hemolysin listeriolysin O (LLO), which is most active at the acidic conditions (pH 5.5) of the vacuole, to confer the lysis of the phagosome membrane [54, 55]. Once the bacteria escape into the cytoplasm, they start to replicate while making use of specific transporter systems to gain carbohydrates from the host cell [56].

At the same time the pathogen is released from the phagosome, it induces the expression of ActA, a protein that triggers the nucleation and polymerization of host globular g-actin into f-actin filaments. The polarized polymerization of actin leads to a propulsive force that propels the bacteria through the cytoplasm and occasionally into the cytoplasma membrane of neighboring cells. The resulting pseudopods or “listeriapods” are then taken up (endocytosed) by the adjacent cells, thus, promoting cell-to-cell spread of the pathogen from on cell to another. The bacteria entrapped within the double membrane of the newly infected vacuole are again released by the combined action of LLO and the phospholipases, in this case the product of the *plcB* gene. This invasion mechanism allows *L. monocytogenes *to safely spread through host tissues without leaving the host cytosolic compartment, thereby protected from the host adaptive immune system. This intriguing strategy has been elucidated and thoroughly reviewed elsewhere [1, 32, 56, 57].

## 5. Innate Immune Responses to *Listeria monocytogenes*

After infection within the gastrointestinal tract, immediate immune responses are essential for the control of pathogens, such as *L. monocytogenes*. Activation of the innate immune system is triggered when pathogen-associated molecular patterns (PAMPs) engage pattern recognition receptors (PRRs) on intestinal epithelial cells (IECs) [58]. Despite the given name, PAMPS are actually not restricted to pathogens. They are expressed by all bacteria, invasive pathogens as well as noninvasive commensals. Typical PAMPs include bacterial carbohydrates, such as lipopolysaccharide (LPS), mannose, nucleic acids (both DNA and RNA), peptidoglycan components, lipoteichoic acids, and probably many other molecules, and are able to trigger the innate immune response. Innate immunity to *L. monocytogenes* is primarily mediated by two types of pattern recognition receptors, the Toll-like receptors (TLRs), and the nucleotide-binding oligomerization domain (NOD)-like receptors (NLRs). In addition, there is some experimental evidence for the involvement of scavenger receptors and a TLR-9 independent cytosolic sensor system for bacterial DNA [59]. 

Toll-like receptors (TLRs) are a family of transmembrane glycoproteins, of which 10 members are known to exist in humans, where they are located on the cell surface or within endosomes. Upon recognition of the presence of microbes through sensing pathogen-associated molecular patterns, TLRs can bind any of the 4 known activating adaptors: (i) Myeloid differentiating factor-88 (MyD88), (ii) MyD88 adapter-like (Mal), (iii) TIR domain-containing adapter-inducing IFN-*β* (TRIF), and (iv) TRIF-related adapter molecule (TRAM), whereas sterile-*α* and Armadillo repeat-containing molecule (SARM) negatively regulates TRIF signaling [60]. MyD88 appears to be the key adaptor molecule, because it is required for signaling by all TLRs with only one exception: TLR3 uses TRIF [7]. The binding of the activating adaptors results in the subsequent recruitment of IL-1R, associated kinases (IRAKs) and downstream activation of transcription factors including NF-*κ*B and IFN regulatory factor 3 (IRF3), which in turn induces the proinflammatory cytokines and type I IFNs [60]. 

In the intestinal mucosa, expression and localization of PRRs on IECs and DCs differ significantly from cells of other tissues. Primary human IECs constitutively express TLR3 and TLR5, but only low levels of TLR2 and TLR4. As the location of the TLRs is crucial for their function, TLRs 1, 2, 4, 5, and 6 are expressed on the cell surface to recognize extracellular microbes, whereas TLR3, 7, 8, and 9 are present on premature endosomes [7]. As mentioned above, expression of TLRs on IECs is generally low and some receptors such as TLR5 and TLR9 are located basolaterally, thus possibly preventing an interaction with PAMPs in the intestinal lumen [7]. However, several TLRs such as TLR2, TLR4, TLR5, and TLR9 are expressed on the apical side of the IECs and important for the recognition of molecules from commensal bacteria, which is crucial to trigger innate immune responses that are required to prevent exaggerated adaptive immunity to the intestinal microbiota [61]. This is an important function indicating that low-level recognition by TLRs is necessary for protection from intestinal epithelial injury [62]. 

Toll-like receptor 2 (TLR2) can interact with several specific ligands, including bacterial lipoproteins, lipoteichoic acids of Gram-positive bacteria such as *L. monocytogenes* and yeast zymosan [7]. TLR2 can form heterodimers with TLR1 and TLR6, thereby improving the recognition of the target lipoteichoic acids [63]. TLR2 is expressed on the cell surface of intestinal epithelial cells and its activation by commensal bacteria is thought to play an important role in the maintenance of the integrity of the intestinal epithelial barrier [64]. TLR2 is also expressed within phagolysosomes, thus, *L. monocytogenes* cells that have escaped into the host cell cytoplasm were not detected by TLR2. The importance of TLR2 signaling for early protection against *L. monocytogenes* is, however, inconclusive. Whereas a first study observed that* L. monocytogenes* infected TLR2 deficient mice were as resistant as wild-type mice, a later study using slightly different experimental settings revealed a protective effect of TLR2 during early *L. monocytogenes* infection [65, 66].

Toll-like receptor 5 (TLR5) can bind to a protein motif common to the flagellin protein making up the flagella from many bacteria, such as *L. monocytogenes*. TLR5 activation induces NF-*κ*B and stimulates TNF production, suggesting that TLR5 may serve as a general alarm system, when the gastrointestinal barrier is compromised by a broad spectrum of motile bacteria. However, activation of TLR5 located on the apical surface of IECs by flagellin leads to an increase in the expression of antiapoptotic genes. This correlates with the observed protective effects of TLR5 signaling in epithelial homeostasis and may suggest that under physiological conditions flagellin ligation of TLR5 located on the apical surface of IECs does not exert an inflammatory response. In contrast, flagellated bacteria that interact with basolateral TLR5 signal an invasion of the epithelium by bacteria, and therefore induce a strong proinflammatory response [7].

On the other hand, flagellin-deficient* L. monocytogenes* revealed no significant differences in virulence in infection experiments. This observation suggests that TLR5 mediated signaling may not be essential for pathogenesis and adaptive immunity after an *L. monocytogenes* infection in immunized animals [67]. 

Toll-like receptor 9 (TLR9) recognizes the CpG motifs present in bacterial DNA. In immune cells, TLR9 is exclusively localized in the endosomes. In the intestine, TLR9 was shown to be located on both, the apical and the basolateral surface of IECs. Upon activation of TLR9, I*κ*B*α* is degraded and NF-*κ*B is activated, again resulting in a proinflammatory response. In contrast, stimulation of apical TLR9 led to the accumulation of polyubiquitinated I*κ*B*α* in the cytoplasm, preventing NF-*κ*B activation and inflammation [7]. Currently, there is no clear evidence that TLR9 actually contributes to the control of *L. monocytogenes* infection; further clarification may be obtained by animals models deficient in TLR9 [6]. 

In conclusion, the available experimental evidence suggests that TLR2 is the most relevant TLR for recognition of *L. monocytogenes* cells. However, as IECs show TLR2 commensal ligand-induced activation, TLR2 is also considered to play an important role in maintaining the integrity of the intestinal epithelial barrier [64]. This view is further supported by the observation of an increased expression of both TLR2 and TLR4, in a neonatal rat model of necrotizing enterocolitis-induced mucosal injury, suggesting that TLR2 may promote intestinal inflammation under circumstances where the epithelial barrier has been compromised [68]. In conclusion, there is no doubt that TLR signaling plays an important role for maintaining the integrity and function of the intestinal epithelium during invasion by *L. monocytogenes*.

NOD-like receptors (NLRs) are a group of intracellular pattern recognition receptors, which are structurally composed of an N-terminal effector domain that can comprise caspase recruitment domains (CARDs) like the NODs, or a pyrin domain as in the case of NALPs (NAcht-, Leucine-rich repeat, and Pyrin domain-containing proteins). Known members of the NLR family are NOD1, NOD2, NALP1, NALP3, neuronal apoptosis inhibitory protein-5, and the ICE protease activating factor (IPAF) [69, 70]. In humans, twenty-three NLRs have been identified so far, while in mice 34 NLRs are known [71]. The NLRs are critical for mucosal innate immunity as sensors of microbial components and cell injury in the cytoplasm [72]. They mediate proinflammatory signals through activation of caspase-1 and NF-*κ*B. Activation of caspase-1 leads to cleavage and activation of proinflammatory cytokines, such as IL-1*β* and IL-18, as well as to apoptotic cell death. Both NOD1 and NOD2 are important for the innate immune response against *L. monocytogenes,* because they represent intracellular sensors of bacterial peptidoglycan components that are thought to enter cells by endocytosis through clathrin-coated pits [73]. While NOD1 is ubiquitously expressed in adult human tissues, NOD2 is expressed only in leukocytes, DCs, and epithelial cells. Activation of NOD1 and NOD2 results in the translocation of NF-*κ*B and mitogen-activated protein kinase into the nucleus, to up-regulate the transcription of proinflammatory genes and mediate antibacterial effects by the up-regulation of another group of small antibacterial peptides, the defensins [7]. 

The nucleotide-binding oligomerization domain 1 (NOD1) recognizes a diaminopimelic acid-containing dipeptide or tripeptide molecule generated by lysozyme action on the peptidoglycan of many Gram-negative and Gram-positive bacteria, including* L. monocytogenes* [74].

The nucleotide-binding oligomerization domain 2 (NOD2) is activated by muramyl dipeptide (MDP), which is another degradation product of the peptidoglycan produced by lysozyme and other (bacterial) peptidoglycan hydrolases. In intestinal Paneth cells, NOD2-mediated signaling is important for the expression of antimicrobial peptides, the cryptidins, which are able to disrupt the membrane function of most bacteria. NOD2-deficient mice revealed an abnormal development and function of Peyer's patches resulting in increased translocation of microbes across Peyer's patches, and increased concentrations of cytokines such as TNF-*α*, IFN-*γ*, IL-12, and IL-14 [75]. As a consequence, NOD2-deficient mice are highly susceptible to* L. monocytogenes *infection via the oral route, but normally susceptible to intravenous challange [76]. This observation demonstrates the importance of NOD2 signaling to prevent infection of the intestinal mucosa by inducing antimicrobial defensins that play an important role in *in vivo* defence against pathogens [77]. The intestinal P glycoprotein also seems to be important for host protection against *L. monocytogenes* GIT infection, most likely by inhibiting absorption of the pathogen into enterocytes [78].

Several NLRs, together with caspase-1, form proinflammatory multiprotein complexes termed the “inflammasomes”. After activation, the molecules assemble and lead to multimerization of the adaptor molecule apoptosis-associated speck-like protein containing a C-terminal caspase recruitment domain (ASC). The signaling cascade results in the processing and secretion of mature IL-1*β* and IL-18, which are mediators for the activation of innate and adaptive immune responses [6]. NLR family members known to form inflammasomes comprise NALP1, NALP2, NALP3, and NALP4.

NALP3 forms an inflammasome complex with ASC, cardinal, and procaspase-1 [79]. The NALP3 pathway is known to be activated by *L. monocytogenes *infection, although the specific ligands that activate NALP3 remain unknown. On the other hand, *L. monocytogenes *DNA in the host cell cytoplasm is known to act as a ligand for a hitherto unknown PRR that mediates induction of IFN-*β* through activation of interferon regulatory factor 3 (IRF3) [80]. Interestingly, cytosolic *L. monocytogenes* actively increase NF-*κ*B activity by expression of the virulence factors listeriolysin O (LLO) and internalin B (InlB). This strategy leads to an increased proinflammatory response and recruitment of immune cells to the site of infection. An interesting suggestion is that the increased response actually promotes spread of intracellular pathogens, by recruiting more host cells, which can serve as potential vehicles for the pathogen [81]. Furthermore, *L. monocytogenes* induces expression of type I interferon (IFN-*αβ*) that are known to be essential for the immune system to clear viral pathogens. However, in contrast to the protective effect to virus infections, in the case of *L. monocytogenes* the IFN-*αβ* induction results in an increase in host susceptibility to the pathogen [82]. The observed benefit might be due either to direct enhancement of bacterial growth, or more likely, to down-modulation of a part of the immune response that plays an important role in controlling bacterial growth. The latter would be supported by the observation that induction of IFN-*αβ* enables *L. monocytogenes* to suppress macrophage activation by IFN-*γ* [82]. It was also shown that early during *L. monocytogenes* infection type I interferons induce T cell apoptosis, resulting in greater IL-10 secretion by phagocytic cells which in turn leads to dampening the innate immune response [83].

Autophagy contributes to innate immune defense against various intracellular bacterial pathogens [84]. When autophagy was discovered, it was thought to serve as a pathway for recycling of intracellular organelles and cytoplasmic constituents as part of cellular homeostasis [6]. For this purpose, a double membrane vacuole is formed around the target object in the cytoplasma. This vacuole is then directed to the lysosome pathway, resulting in degradation of the vacuolar content. Similarly, intracellular bacteria can be targeted and destroyed, as it is well known for intracellular pathogens such as *Salmonella*, Group A *Streptococcus*, or *Mycobacterium tuberculosis* [85–87]. However, other bacteria are able to evade or even exploit autophagy during infection [88]. Pathogens adapted to persistence in the cytoplasm, such as *Listeria monocytogenes* and *Shigella flexneri*, have evolved mechanisms to avoid autophagy [89, 90].

For *Salmonella* and *Toxoplasma,* it was shown that the damaged vacuole itself triggers autophagy of the pathogens [85, 91]. However, for *Listeria monocytogenes *infection in *Drosophila melanogaster,* it was demonstrated that a peptidoglycan-recognition protein, acting as an intracellular PRR, plays an essential role for autophagy protection from the pathogen [92]. Results from previous studies indicated that *L. monocytogenes* deploy several mechanisms to evade from autophagy [90, 93, 94], and it was speculated whether this effect is mainly due to actin-based motility, or due to masking of the bacterial cell [93]. An earlier study suggested that active bacterial protein synthesis is required to escape from autophagy in macrophages [90], and results from a more recent study led to the assumption that bacterial phospholipases (PI-PLC and PC-PLC) may play another role [93]. How the bacterial Plc enzymes are involved is not entirely clear, but they are thought to either mediate escape from the autophagosome or prevent their formation [95]. However, in a recent study, Yoshikawa et al. [96] clearly showed that during primary infection *L. monocytogenes *avoids autophagy by disguising itself as a host organelle. Due to the ability of ActA to recruit host cell cytoskeleton proteins such as the Arp2/3 complex and VASP, the pathogens avoid ubiquitination and p62 accumulation. Moreover, it was demonstrated that lack of actin-based motility alone is not sufficient to escape from autophagy. 

Although *L. monocytogenes *was previously thought to primarily reside in the cytoplasm, a recent study described the presence of variant *L. monocytogenes* forms that replicate in macrophages, inside large, LAMP1-positive vacuoles designated as spacious *Listeria*-containing phagosomes (SLAPs) [97]. The formation of SLAPs seems to be promoted by inefficient LLO activity that is not sufficient for bacterial escape from phagosomes, which triggers an autophagic response to the damaged phagosome. On the other hand, the LLO leads to disruption of the proton gradient, thereby preventing fusion with lysosomes. Within the SLAPs the bacteria are able to replicate, but the replication rate is low compared to the cytoplasm [97, 98]. The reason for the impaired LLO expression of bacteria in SLAPs is unknown. However, it is known that LLO activity is low in LAMP-1 or alkaline compartments, which are the characteristics of SLAPs [98, 99]. Moreover, function of LLO can be impaired by innate immune responses, such as reactive oxygen and nitrogen intermediates, and cathepsin D [100, 101]. Because the maturation of phagosomes is quite heterogeneous, the bacteria stuck in SLAPs may be effected by other host innate factors, compared to the bacteria that managed to escape from the phagosome [95].

## 6. Adaptive Immune Responses to *Listeria monocytogenes*

Adaptive immune responses follow the initial innate immune responses and dendritic cells (DCs) represent an important link between the two immunological pathways [102]. DCs respond to different pathogens and initiate the appropriate type of T cell response needed to control the infection. In response to *L. monocytogenes *infection, DCs are critical in priming the T cell response, since mice depleted of DCs are unable to generate a CD8 T cell response [103]. Due to the primarily intracellular localization of *L. monocytogenes*, CD4 and CD8 T cells mediate most of the adaptive immune response, and are crucial for long-term immunity after initial *L. monocytogenes *infection. Other cell subsets may contribute by influencing the CD4 and CD8 T cell responses. Whereas innate immune cells are important for initial control of *L. monocytogenes *infection, T cells are required for final clearance of the pathogen. Almost any cell type that harbors *L. monocytogenes *in the cytoplasm can process the proteins secreted from the pathogen, by degradation and subsequent loading on MHC class I molecules, in order to present them on the cell surface to CD8 T cells. Only professional antigen-presenting cells (APCs) can present antigens derived from lysosomal degradation via the MHC class II pathway to CD4 T cells [6]. The CD8 T cells mediate the anti-*Listeria* immunity by two synergistic mechanisms: first, by secretion of IFN-*γ* to activate macrophages; secondly, by lysis of infected cells via perforin and granzymes, leading to the exposure of intracellular bacteria to the activated macrophages [104]. IFN-*γ* is known to be essential for host resistance to intracellular pathogens such as *L. monocytogenes,* as it mediates the activation of resting macrophages that more efficiently restricts the multiplication of intracellular pathogens and promotes long-term protective cellular immunity [105]. 

The role of CD4 T cells in the course of the control of *L. monocytogenes *infection is much less well understood. *L. monocytogenes *induces a strong T-helper type 1 response and, similar to CD8 T cells, CD4 T cells also secrete IFN-*γ*. The strong CD8 and CD4 T cell responses results in a stable population of memory T cells specific for *L. monocytogenes* [6]. 

In the intestine, NKT cells (lymphocytes expressing both NK and T cell markers) play an important role in the control of early infection with *L. monocytogenes* [106]. It was shown that processing and presentation of listerial antigens is mediated by a distinct population of DCs, and strong costimulation is necessary for the development of a local antigen-specific T cell response in the intestinal mucosa. This strong costimulation seems to be required to activate appropriate antilisterial T cells and to surmount tolerance within the generally immunosuppressive intestinal milieu [107]. 

In general, adaptive immune responses in the intestine are characterized by high numbers of IgA producing plasma cells, regulatory T cells, and IL-17 producing T cells whose development is closely linked to factors produced by PRRs expressing IECs, DCs, and macrophages. This indicates that PRR mediated recognition of ligands produced by commensal bacteria is involved in positive and negative regulation of both, innate and adaptive immunity in the intestine [7].

## 7. *Listeria monocytogenes* L-Forms

L-forms are protoplast-like variants of bacteria that lost their ability to maintain a rigid cell wall. They have been first described at the beginning of the last century and were reported for many bacterial species. After their discovery, they have been intensively studied using numerous approaches [108]. However, due to the fastidious nature of L-form bacteria and experimental difficulties using old-fashioned techniques, these studies mainly focused on morphology and physiology of L-form cells and often led to inconclusive data. This was also the case for L-forms of *L. monocytogenes* [109]. It was only recently that L-form research experienced a renaissance, due to new experimental systems and the application of molecular biology and state-of-the-art imaging techniques [110–112]. In a recent study, it was demonstrated that stable L-forms of *L. monocytogenes* are viable bacteria that are not only able to survive, but also able to replicate and multiply using a unique, previously unknown mechanism [110]. Thus, L-forms are unlikely to be just artifacts found under laboratory conditions, but seem to represent a pre-programmed, alternative phenotype of bacterial life. Of particular interest is the observation that *L. monocytogenes* L-forms are able to persist within macrophages, suggesting that they retain at least a part of their pathogenetic traits (Schnell et al., unpublished data). Previous results from tissue culture studies already suggested that L-forms may be able to persist within eukaryotic cells for various time periods [113, 114]. Clinical case reports about the isolation of cell wall-deficient variants in cases of persistent and recurrent bacterial infection also suggested that L-forms may serve as cryptic agents of disease in a variety of human infectious diseases [114–118]. Subsequent reversion to parental forms may lead to a damage of the host cells. In the case of L-forms, when the bacteria have completely shed their cell walls, several proteins that represent important markers for the human immune system are also lost. Therefore, the immune system may no longer be able to discern and recognize these bacteria cells. Lack of the cell wall as an important target for antibiotic treatment represents a further threat, due to the ineffectiveness of cell-wall active drugs such as *β*-lactams and cephalosporins on L-form cells [110, 119].

## 8. The Possible Role of *Listeria* in Inflammatory Bowel Disease

Inflammatory bowel disease is a collective term for Crohn's disease and ulcerative colitis, both immune-mediated diseases of the gastrointestinal tract which can develop in genetically susceptible individuals [120]. A potential role of *L. monocytogenes *in the pathogenesis of inflammatory bowel disease (IBD) has been suggested, because interference of the pathogen with NOD2-based signaling [121], and variations of NOD2/CARD15 have been shown to represent a risk factor for Crohn's disease [122]. Especially noteworthy seems the presence of *L. monocytogenes* at the site of colon perforation in a patient with fulminant ulcerative colitis [123]. Another study, however, reported an equal prevalence of* L. monocytogenes* in patients suffering from IBD and non-IBD control patients [124], suggesting a more common occurrence of the pathogen in the gastrointestinal environment. Together with the observation of a low prevalence of *L. monocytogenes* in biopsies from IBD patients the available data do not yet support a role of *L. monocytogenes* in IBD [120]. However, there is still a lot to be done to unravel any potential indirect involvement of *L. monocytogenes *in the pathogenesis of IBD.

*Listeria monocytogenes *has been employed for decades as a model organism to study host-pathogen interactions and immune responses against intracellular pathogens [125]. The many studies provide significant insight into how *L. monocytogenes *interacts on host mucosal surfaces of the human gastrointestinal tract with the immune system that triggers the antibacterial immune responses. Despite the vast amount of knowledge gathered on the host-pathogen interactions and the bacterial adaptations to mammalian host, it was only recently that *L. monocytogenes *was found to be responsible for induction of local mucosal inflammation in immuno-competent individuals, resulting in febrile gastroenteritis [4]. Hence, the availability of new and improved animal models, such as a humanized mouse model [49], will be an important prerequisite to improve the investigation of the gastrointestinal phase of *L. monocytogenes* infection, in order to further enhance our understanding of the interaction and the interplay of the pathogen with the host intestinal mucosa.

## Figures and Tables

**Table 1:** Outbreaks of gastroenteritis due to *Listeria monocytogenes. *

Year of outbreak	Number of cases	Serotype	Implicated source	Reference
1993	18	1/2b	Rice salad	[41]
1994	45	1/2b	Chocolate milk	[30]
1997	1566	4b	Cold-corn-and-tuna salad	[31]
1998	5	1/2a	Cold smoked trout	[42]
2000	32	1/2	Corned beef and ham	[43]
2001	16	1/2a	Delicatessen meat	[44]
2001	48	1/2a	Cheese	[45]
